# Supplementary material for: End Sequence Analysis Toolkit (ESAT) expands the extractable information from single-cell RNA-seq data
Source: Genome Res. 2016 Oct;26(10):1397–410. doi: 10.1101/gr.207902.116 (PMC5052061; doi:10.1101/gr.207902.116)
Supplement: Supplemental Material [file supp_gr.207902.116_Supp_Materials.tar.gz › ESAT-esat_v0.1/doc/html/classbroad_1_1pda_1_1differential_expression_1_1_run_differential_expression.html]

ESAT: broad.pda.differentialExpression.RunDifferentialExpression Class Reference


ESAT


- Main Page
- Related Pages
- Packages
- Classes
- Files

- Class List
- Class Index
- Class Hierarchy
- Class Members

All Classes Namespaces Files Functions Variables Enumerator Pages

- broad
- pda
- differentialExpression
- RunDifferentialExpression

Public Member Functions |
Static Public Member Functions |
List of all members

broad.pda.differentialExpression.RunDifferentialExpression Class Reference

Collaboration diagram for broad.pda.differentialExpression.RunDifferentialExpression:

[legend]

|  |  |
| --- | --- |
| Public Member Functions | |
| MatrixWithHeaders | getResults () |
|  | |
| void | writeResults (String outFile) throws IOException |
|  | |
|  | RunDifferentialExpression (File gctFile, File experimentInfoFile, File chipFile, String save, boolean preprocess, double alpha, int perm) throws IOException, ParseException |
|  | |
|  | RunDifferentialExpression (MatrixWithHeaders data, Collection< String > group1, Collection< String > group2, int numPerm, double alpha) |
|  | |
|  | RunDifferentialExpression (MatrixWithHeaders data, Map< String, Collection< String >> groups, int numPerm, double alpha) throws IOException |
|  | |
| MatrixWithHeaders | getGenesPassingFDR (double alpha) |
|  | |
| MatrixWithHeaders | getFDR () |
|  | |

|  |  |
| --- | --- |
| Static Public Member Functions | |
| static void | main (String[] args) throws IOException, ParseException |
|  | |

## Constructor & Destructor Documentation

|  |  |  |  |
| --- | --- | --- | --- |
| broad.pda.differentialExpression.RunDifferentialExpression.RunDifferentialExpression | ( | File | *gctFile*, |
|  |  | File | *experimentInfoFile*, |
|  |  | File | *chipFile*, |
|  |  | String | *save*, |
|  |  | boolean | *preprocess*, |
|  |  | double | *alpha*, |
|  |  | int | *perm* |
|  | ) |  | throws IOException, ParseException |

Here is the caller graph for this function:

|  |  |  |  |
| --- | --- | --- | --- |
| broad.pda.differentialExpression.RunDifferentialExpression.RunDifferentialExpression | ( | MatrixWithHeaders | *data*, |
|  |  | Collection< String > | *group1*, |
|  |  | Collection< String > | *group2*, |
|  |  | int | *numPerm*, |
|  |  | double | *alpha* |
|  | ) |  |  |

Parameters
:   |  |  |
    | --- | --- |
    | data | Data set in which to find differentially expressed genes |
    | group1 | The list of samples (columns in the data matrix) of group 1 |
    | group2 | The list of samples (columns in the data matrix) of group 2 |
    | numPerm | Number of permutations to perform. Specify 0 to perform the maximal number of permutations |
    | alpha | Significance level |

Exceptions
:   |  |  |
    | --- | --- |
    | IOException |  |

|  |  |  |  |
| --- | --- | --- | --- |
| broad.pda.differentialExpression.RunDifferentialExpression.RunDifferentialExpression | ( | MatrixWithHeaders | *data*, |
|  |  | Map< String, Collection< String >> | *groups*, |
|  |  | int | *numPerm*, |
|  |  | double | *alpha* |
|  | ) |  | throws IOException |

## Member Function Documentation

|  |  |  |  |  |
| --- | --- | --- | --- | --- |
| MatrixWithHeaders broad.pda.differentialExpression.RunDifferentialExpression.getFDR | ( |  | ) |  |

|  |  |  |  |  |  |
| --- | --- | --- | --- | --- | --- |
| MatrixWithHeaders broad.pda.differentialExpression.RunDifferentialExpression.getGenesPassingFDR | ( | double | *alpha* | ) |  |

Here is the call graph for this function:

|  |  |  |  |  |
| --- | --- | --- | --- | --- |
| MatrixWithHeaders broad.pda.differentialExpression.RunDifferentialExpression.getResults | ( |  | ) |  |

|  |  |  |  |  |  |
| --- | --- | --- | --- | --- | --- |
| static void broad.pda.differentialExpression.RunDifferentialExpression.main | ( | String[] | *args* | ) | throws IOException, ParseException |

static

Here is the call graph for this function:

|  |  |  |  |  |  |
| --- | --- | --- | --- | --- | --- |
| void broad.pda.differentialExpression.RunDifferentialExpression.writeResults | ( | String | *outFile* | ) | throws IOException |

---

The documentation for this class was generated from the following file:

- java/broad/pda/differentialExpression/RunDifferentialExpression.java


---

Generated on Fri Aug 15 2014 15:10:59 for ESAT by  

 1.8.7
